# *Mrakia panshiensis* sp. nov. a new member of the Cystofilobasidiales from soil in China, and description of the teleomorphic-stage of *M. arctica*

**DOI:** 10.3897/mycokeys.74.53433

**Published:** 2020-10-20

**Authors:** Kai-Hong Zhang, Cheng-Feng Shi, Chun-Yue Chai, Feng-Li Hui

**Affiliations:** 1 School of Life Science and Technology, Nanyang Normal University, Nanyang 473061, China Nanyang Normal University Nanyang China

**Keywords:** *
Cystofilobasidiales
*, *
Mrakiaceae
*, one new species, soil-inhabiting yeasts, taxonomy

## Abstract

In a study on the fungal diversity in Northeast China, twelve yeast isolates were obtained from soils collected in three provinces, Helongjiang, Jilin and Liaoning. Morphological assessment and phylogenetic analyses of the nuc rDNA internal transcribed spacer (ITS) region and the D1/D2 domains of the nuc 28S rDNA (nuc 28S) gene of the 12 cultures placed them in the genus *Mrakia*, namely *Mrakia aquatica*, *Mrakia arctica*, *Mrakia frigida*, *Mrakia gelida* and *Mrakia robertii*. A total of three isolates represented a hitherto undescribed species, which is described here as *M. panshiensis***sp. nov.** (MB 834813). The species *M. panshiensis***sp. nov.** shares several morphological characters with *M. niccombsii*, *M. aquatica*, *M. fibulata* and *M. hoshinonis*. These species can be distinguished based on physiological traits and pairwise rDNA sequence similarities. The study also describes for the first time the formation of teliospores by previously described *M. arctica*.

## Introduction

The first *Mrakia* species was described as a new species *Candida curiosa* from a frozen food (Komagata and Nakase 1965). Subsequently, three new *Candida* species, *Candida frigida*, *C. gelida* and *C. nivalis*, were obtained from soil samples in Antarctic and Greenland (Di Menna 1966). Fell et al. (1969) observed that these *Candida* species exhibit a heterobasidiomycetous lifecycle, and hence reclassified them in the basidiomycetous genus *Leucosporidium*. Yamada and Komagata (1987) reclassified these taxa to a new genus once again, *Mrakia* (Mrakiaceae, Cystofilobasidiales), as *M. frigida*, *M. gelida*, *M. nivalis* (now considered a synonym of *M. frigida*), and *M. stokesii* (now considered a synonym of *M. gelida*), since these isolates have a CoQ8 system while other *Leucosporidium* species have CoQ9 or CoQ10 (Tsuji et al. 2019). Since then, seven further species have been described, namely *M. curviuscula* (now classified in the genus *Krasilnikovozyma*) (Bab’eva et al. 2002; Liu et al. 2015), *M. psychrophila* (Xin and Zhou 2007), *M. robertii*, *M. blollopis* (Thomas-Hall et al. 2010), *M. arctica* (Tsuji et al. 2018), *M. hoshinonis* (Tsuji et al. 2019), and *M. fibulata* (Yurkov et al. 2020). In addition to sexual species classified in the genus *Mrakia*, the order Cystofilobasidiales (Fell et al., 1999) included anamorphic species that were originally classified in the genus *Cryptococcus* and later in the anamorphic genus *Mrakiella* (Margesin and Fell 2008). With the end of dual naming of pleomorphic fungi, asexual species of the genus *Mrakiella* (*M. aquatica*, *M. cryoconiti*, and *M. niccombsii*) were transferred in the genus *Mrakia*, which was emended to include asexual states (Liu et al. 2015). In the same study, *M. curviuscula* was excluded from the emended genus *Mrakia* because this species built a distinct clade together with the anamorph species *Krasilnikovozyma* (formerly *Cryptococcus*) *huempii* and a few other closely related species (Liu et al. 2015). Recently, based on molecular and morphological analyses, two new taxa, namely *M. montana* and *M. stelviica*, were described in *Mrakia* (Turchetti et al. 2020). Thus, twelve species are presently accepted in the genus *Mrakia*.

The genus *Mrakia* has been demonstrated to be a monophyletic group based on phylogenetic analyses of sequences of D1/D2 domains of nuc 28S rDNA and ITS region (Fell et al. 2000; Scorzetti et al. 2002; Margesin and Fell 2008), and the multi-gene phylogenetic analysis that limited to its current circumscription, the *Mrakia* clade recognised in Liu et al. (2015). This genus is characterized by the formation of true hyphae, pseudohyphae, lack of basidiocarps, the ability to utilize nitrate and nitrite, and have coenzyme Q-8, Q-9 or Q-10 system (Liu et al. 2015). Asexual reproduction is by polar budding; sexual reproduction is by formation of teliospores that occur terminally and intercalarly (Fell and Margesin 2011; Fell 2011). *Mrakia* species share a remarkable adaptation to low temperatures, in some case even below freezing point (Buzzini et al. 2018; Yurkov et al. 2020), by producing diverse extracellular cold-active enzymes such as lipases, amylases proteases, pectinases, cellulases, chitinases, and ligninolytic enzymes (Tasselli et al. 2017; Tsuji et al. 2018, 2019). These abilities have been applied to wastewater treatment and bioethanol production and low temperatures fermentation (Tsuji et al. 2013a, b; De Francesco et al. 2018).

During a study on the fungal diversity in Northeast China, in the provinces of Helongjiang, Jilin and Liaoning, several soil-inhabiting yeasts were cultured. Based on the identification sequences of the D1/D2 domains of the nuc 28S rDNA (nuc 28S) gene, these isolates were assigned to the genus *Mrakia*. After a detailed phylogenetic analysis of a concatenated alignment of sequences of the complete ITS region and D1/D2 domains as well as a micro-morphological study, the yeasts were identified as six *Mrakia* species, including one potential novel species. This novel species is described here as *Mrakia panshiensis* sp. nov.. Additionally, using a culture isolated in the present study, we describe the formation of teliospores by previously described *M. arctica*.

## Materials and methods

### Sample collection, morphological studies and isolation

Biodiversity assessment of soil yeasts was performed in three provinces of China, Helongjiang, Jilin and Liaoning. Forty-five soil samples were collected from the Lianhuashan National Forest Park (approximate GPS coordinates: 43°92'N, 125°71'E) in Panshi city, Jilin Province. The climate of this area is temperate, with an annual precipitation between 650 to 720 mm, and an average temperature ranging from –3 to 10 °C. Thirty-five soil samples were collected from the Maoershan National Forest Park (approximate GPS coordinates: 45°28'N, 127°58'E) in Yanji city, Heilongjiang Province. The forest park is characterized by temperate and humid climates, with annual precipitation between 400 to 650 mm, and an average temperature from –2 to 6 °C. Forty soil samples were collected from the Tianhuashan National Forest Park (approximate GPS coordinates: 41°09'N, 124°62'E) in Dandong city, Liaoning Province. The climate of this area is temperate and humid, with annual precipitation between 800 to 1100 mm, and an average temperature from 5 to 7 °C. Three studied locations are situated in Northeast China. The sampling sites were forested areas in the mountain cold broad-leaved and mixed forest biomes; they have a long and very cold winter.

Soil samples were collected in September 2018 as following: samples were taken from the top 15 cm layer, avoiding stones and organic materials as much as possible, and transferred into two 20 ml plastic tubes with screw-lids. Within 1 h after the sampling, samples were cooled and stored at 4 °C until analysis. Yeast strains were isolated following the method described by Groenewald et al. (2018). Specifically, an aliquot of each sample (1 g) was subjected to a serial dilution in sterile distilled water and mixed by vortex for 30 s. Subsequently, 0.1 ml of soil suspension ranging from 10^−1^–10^−4^ (w/v) were spread on four plates containing yeast extract-malt extract (YM) agar (1% glucose, 0.5% peptone, 0.3% yeast extract, 0.3% malt extract and 2% plain agar; pH 5.4) supplemented with 0.02% chloramphenicol. The plates were incubated at 10 °C for up to three weeks and examined every three days. Colonies were differentiated into macro-morphological types using dissection microscopy by colony morphology and color, counted and two representatives of each colony morphotype per sample were purified by streaking at least twice, and then stored in 15% glycerol at −80 °C.

The morphological observations and metabolic tests were performed according to the standard procedures described by Kurtzman et al. (2011). Assimilation tests of carbon and nitrogen sources were performed in liquid media at 15 °C. Starved inocula were used in nitrogen and vitamin assimilation tests, and the results were read after 5 and 21 days of incubation. Induction of the sexual stage was tested by incubating cultures singly or crossed pair-wise on cornmeal (CM) agar, YM agar or 5% malt extract (5% ME) agar at 10 °C for 2 months (Yurkov et al. 2020). Photomicrographs were taken with a Leica DM5000B microscope (Leica). Culture growth was examined at 1–30 °C in YM liquid culture and on YM agar plates.

### DNA extraction, PCR amplification and sequencing

Genomic DNA was extracted from the cultures using the Ezup Column Yeast Genomic DNA Purification Kit, according to the manufacturer’s protocol (Sangon Biotech, Shanghai, China). The nuc rDNA internal transcribed spacer (ITS) region was amplified using the primer pairs ITS1/ITS4 (White et al. 1990). The D1/D2 domain of the nuc 28S rDNA gene was amplified using the primer pair NL1/NL4 (Kurtzman and Robnett 1998). Amplification of ITS and nuc 28S rDNA gene was accomplished by an initial step of 2 min at 95 °C, followed by 35 cycles of 30 s at 95 °C, 30 s at 51 °C (for ITS and nuc 28S rDNA, respectively) and 40 s at 72 °C, with a final extension of 10 min at 72 °C (Liu et al. 2016). PCR products were directly purified and sequenced by Sangon Biotech Inc. (Shanghai, China). We determined the identity and accuracy of newly-obtained sequences by comparing them to sequences of isolates of the genus *Mrakia* in GenBank. Sequences were assembled using BioEdit V7.0.9.0 (Hall 1999). Newly-obtained sequences were submitted to GenBank (https://www.ncbi.nlm.nih.gov/genbank/) under accession numbers provided in Table 2.

### Phylogenetic analysis

The ITS region, the nuc 28S rDNA gene and the concatenated alignment of ITS and nuc 28S rDNA sequences were employed to delimit species of the genus *Mrakia*. The selection of strains and species within the genus *Mrakia* followed most recent descriptions of species in the genus, Yurkov et al. (2020), Tsuji et al. (2017, 2019). The ITS region and the nuc 28S rDNA gene sequences were aligned with the MAFFT program ver. 7.273 (Katoh and Standley 2013) using the G-INS-I algorithm. The alignments were deposited in TreeBASE (Submission ID: S26876 and S26877; www.treebase.org), respectively. Major insertions and ambiguous regions were identified and eliminated with Gblocks version 0.91b (Castresana 2000) using a relaxed selection (minimum number of sequences for a conserved position = 15, minimum number of sequences for a ﬂank position = 15, maximum number of contiguous non-conserved positions = 8, minimum length of a block = 5 and allowed gap positions = ‘with half’), following Talavera and Castresana (2007). The edited alignment was deposited at TreeBase (submission ID S26878 and S26879; www.treebase.org). Maximum likelihood (ML) with an HKY+G+I model was performed using MEGA 7 (Kumar et al. 2016). Bayesian inference (BI) was constructed using MrBayes 3.2.5 (Ronquist et al. 2012) with a GTR+I+G model and 5,000,000 generations, two independent runs, and four chains. The other parameters were set as the default values. We discarded 25% of these trees, and remaining trees were used to compute a 50% majority rule consensus tree to estimate posterior probabilities. A bootstrap analysis with 1000 replicates was performed to estimate the confidence of the tree nodes and a bootstrap percentage (BP) of 50% or Bayesian posterior probability (BPP) of 0.9 was considered supportive in all constructed trees in this study.

## Results

### Phylogenetic analyses

A total of 135 yeast isolates were isolated from the 120 soil samples. Twelve isolates from 12 different soil samples built a rather uniform group based on a careful examination of morphological characteristics. Based on pair-wise sequence similarity comparisons and phylogenetic analyses of the ITS region and the nuc 28S rDNA, these 12 strains were identified as members of the genus *Mrakia*, *Mrakia gelida* (three isolates), *Mrakia robertii* (two isolates), *Mrakia frigida* (one isolate), *Mrakia aquatica* (two isolates) and one isolates was classified as *Mrakia arctica* (Table 1). Three strains represented a potential new species, which is described in detail in the present study. The remaining more than 100 isolates from soils require further identification and characterization.

**Table 1.** Strains isolated in this work and correspondent isolation sources.

Species	Strain	Source	Location
*Mrakia aquatica*	NYNU 18538	Soil and lichen	The Lianhuashan National Forest Park, Jilin Province, China
	NYNU 19451	Soil	The Lianhuashan National Forest Park, Jilin Province, China
*Mrakia arctica*	NYNU 18469	Soil	The Tianhuashan National Forest Park Liaoning Province, China
*Mrakia frigida*	NYNU 1846	Soil	The Tianhuashan National Forest Park Liaoning Province, China
*Mrakia gelida*	NYNU 18513	Soil	The Tianhuashan National Forest Park Liaoning Province, China
	NYNU 1834	Soil and lichen	The Tianhuashan National Forest Park Liaoning Province, China
	NYNU 18473	Soil	The Tianhuashan National Forest Park Liaoning Province, China
*Mrakia panshiensis*	NYNU 18562	Soil	The Lianhuashan National Forest Park, Jilin Province China
	NYNU 18410	Soil	The Maoershan National Forest Park Heilongjiang Province, China
	NYNU 1941	Soil	The Maoershan National Forest Park Heilongjiang Province, China
*Mrakia robertii*	NYNU 18415	Soil	The Lianhuashan National Forest Park, Jilin Province, China
	NYNU 184159	Soil and lichen	The Lianhuashan National Forest Park, Jilin Province, China

**Table 2.** Sequences used in molecular phylogentic analysis. Entries in bold are newly generated for this study.

Species	Strain	ITS	D1/D2
*Mrakia aquatica*	CBS 5443^T^	AF410469	NG_042348
	**NYNU 18538**	**MT126040**	**MT126039**
	**NYNU 19451**	**MT140347**	**MT140348**
*Mrakia arctica*	JCM32070 ^T^	LC222845	LC222845
	**NYNU 18469**	**MK682823**	**MK682821**
*Mrakia blollopis*	CBS 8921^T^	AY038826	NG_057710
*Mrakia cryoconiti*	CBS 10834^T^	AJ866976	KY108575
*Mrakia fibulata*	DSM 103931^T^	MK372216	MK372216
*Mrakia frigida*	CBS 5270^T^	AF144483	NG_042346
	**NYNU 1846**	**MT126288**	**MT133538**
*Mrakia gelida*	CBS 5272^T^	AF144485	AF189831
	**NYNU 1834**	**MT126029**	**MT126028**
	**NYNU 18473**	**MT133535**	**MT133534**
	**NYNU 18513**	**MT133539**	**MT133537**
*Mrakia hoshinonis*	JCM 32575^T^	LC335798	LC335798
*Mrakia niccombsii*	CBS 8917 ^T^	AY029346	NG_060242
*Mrakia panshiensis*	**NYNU 18410**	**MT133553**	**MT133536**
	**NYNU 18562^T^**	**MK682818**	**MK682815**
	**NYNU 1941**	**MT133552**	**MT133554**
*Mrakia psychrophilia*	CBS 10828^T^	EU224267	KY108586
*Mrakia robertii*	CBS 8912^T^	AY038829	AY038811
	**NYNU 18415**	**MT125967**	**MT125965**
	**NYNU 184159**	**MT133533**	**MT133532**
*Mrakia* sp.	strain H2	AY052488	AY052480
	strain H1	AY052487	AY052479
*Mrakia stelviica*	CBS 16461	MT347764	MT347768
*Mrakia montana*	CBS 16462	MT347765	MT347769
*Tausonia pullulans*	CBS 2532^T^	AF444417	NG_042352

Abbreviations: **CBS**: CBS-KNAW Collections, Westerdijk Fungal Biodiversity Institute, Utrecht, The Netherlands; **JCM**: RIKEN BioResource Research Center-Japan Collection of Microorganisms, Takao, Japan; **DSM**: the German Collection of Microorganisms and Cell Cultures, Braunschweig, Germany; **NYNU**: Microbiology Lab, Nanyang Normal University, Henan, China; **T**: type strain.

**Figure 1.**
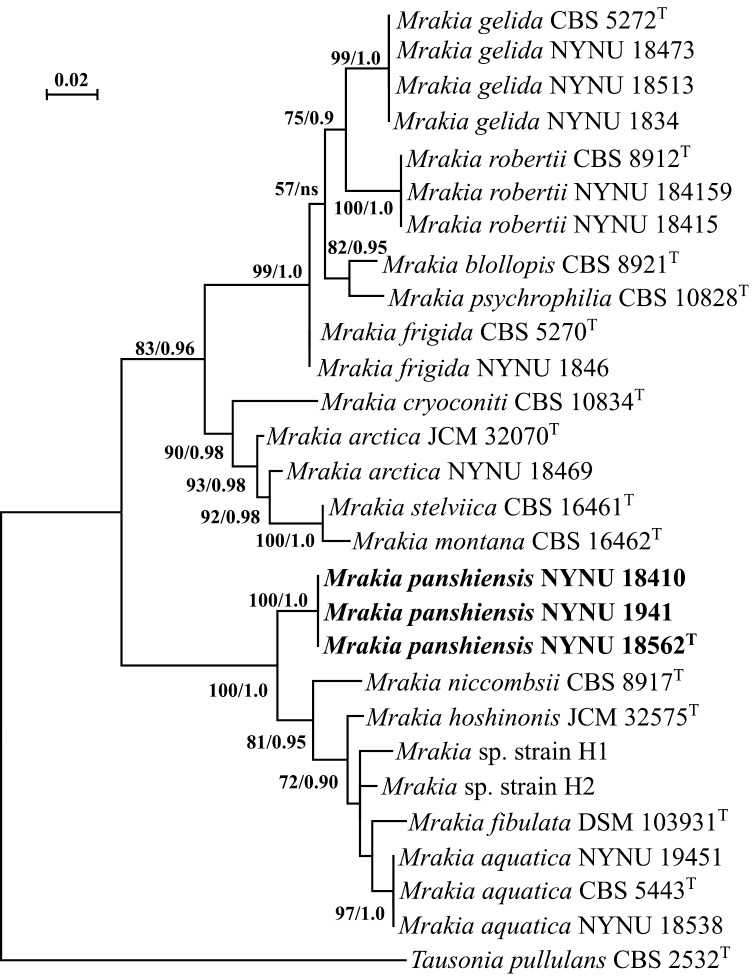
Phylogram inferred from Maximum likelihood analysis of the concatenated ITS and nuc 28S rDNA dataset of taxa in *Mrakia* s.s. *Mrakia panshiensis* strains investigated in this study are highlighted in bold font. *Tausonia pullulans*CBS 2532^T^ was designated as the outgroup. The tree backbone was constructed by maximum likelihood analysis with MEGA7. Bootstrap percentages of maximum likelihood over 50% from 1000 bootstrap replicates and posterior probabilities of Bayesian inference above 0.9 are shown from left on the branches. The scale bar represents 0.02 substitutions per nucleotide.

The three isolates (NYNU 18562, NYNU 1941, NYNU 18410) shared identical sequences of both nuc 28S rDNA and ITS region. In terms of pairwise sequence similarity, the novel species showed a sequence divergence of 1.5% (9 substitutions and 0 gap over 602 bases) in nuc 28S rDNA from the closest relative *M. niccombsii*. The novel species also differed from other members of the *M. aquatica* sub-clade, *M. aquatica*, *M. fibulata* and *M. hoshinonis*, by sequence divergences ranging from 1.7% to 2% (10 to 12 substitutions and 0 to 1 gap over 602 bases) and from 3.3% to 2.5% (11 to 14 substitutions and 4 to 6 gaps over 603 bases), in nuc 28S rDNA and ITS, respectively. The phylogram, based on concatenated alignments of sequences of the the ITS region and nuc 28S rDNA showed that the three isolates represent a novel species in the *Mrakia aquatica* sub-clade, close to *M. niccombsii*, *M. aquatica*, *M. fibulata* and *M. hoshinonis* (Fig. 1). Topologies of phylogenetic trees that were made using alignments of either ITS region or nuc 28S rDNA and inferred from BI and ML algorithms were visually similar and, thus, only ML trees were shown (Suppl. material 1: Figs S1, S2). The combined ITS and nuc 28S rDNA dataset included 28 yeast collections representing 12 species of the genus *Mrakia* with *Tausonia pullulans*CBS 2532^T^ as the outgroup (Fig. 1). The alignment had a total of 1243 characters, of which 1031 characters were constant, 210 were variable and parsimony-uninformative and 114 were parsimony-informative. ML and BI analyses yielded trees which were topologically congruent in terms of species clusters (examined visually). Three strains of the new species formed a well-supported branch basal to other members of the *M. aquatica* sub-clade with high statistical support of 100% BP and 1.0 BPP in ML and BI analyses, respectively. The strain NYNU 18469 clustered with high support with the type strain of *M. arctica* showing 99.8% and 99.3% sequence identities in the nuc 28S rDNA and ITS region, respectively.

## Taxonomy

### Mrakia panshiensis

Taxon classificationFungiCystofilobasidialesMrakiaceae

R.R. Jia & F.L. Hui sp. nov.

BDC4516C-BB30-533D-B4B0-14C60F902FD1

834813

Fig. 2

#### Etymology.

The species name *panshiensis* (N.L. fem. adj.) refers to the geographical origin of the type strain of this species.

#### Description.

**Figure 2.**
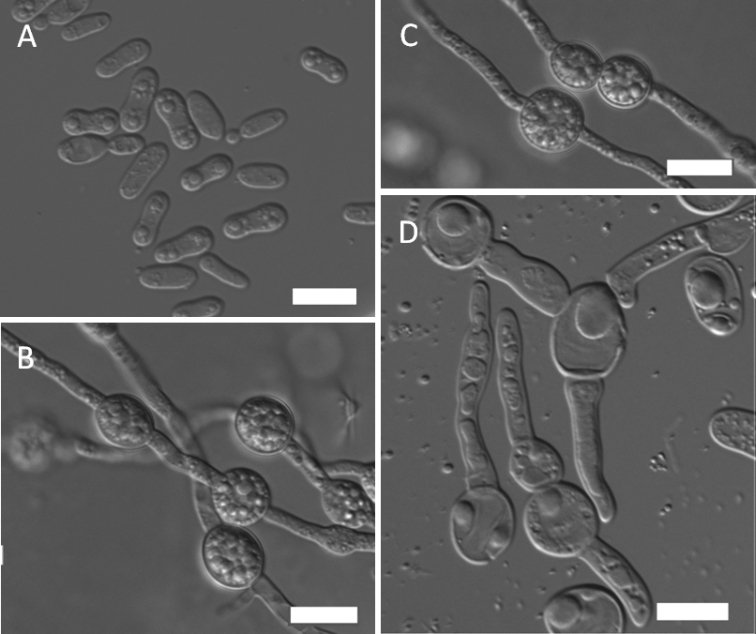
Morphology of *Mrakia panshiensis* (NYNU 18562^T^) **A** budding cells **B** true hyphae with clamp connections and teliospores **C** teliospores in pairs **D** teliospores produced with a bud-like projection. Scale bars: 10 μm.

The physiological profiles of the three strains of the novel species were almost identical. In YM broth after 3 days at 15 °C, the cells are ovoid to elongated (3.5–7 × 3.5–5 µm) and proliferate by polar budding (Fig. 2A). Streak culture for 1 week at 15 °C on YM agar produces colonies that are white to yellowish-cream, round, convex and smooth with an entire margin. After 2 weeks in Dalmau plate culture on CM agar at 15 °C, pseudohyphae and true hyphae are formed. Teliospores were observed after 2 months at 10 °C terminally and intercalarily on the hyphae on CM agar (Fig. 2B). Teliospores are spherical, 8–13 µm in diameter, single and in short chains of 2–4 spores (Fig. 2C). Teliospores may germinate by a bud-like projection (Fig. 2D). The fermentation of sugars is absent. Glucose, galactose, l-sorbose, d-ribose, d-xylose, l-arabinose, l-rhamnose, sucrose, maltose, trehalose, methyl α-d-glucoside, cellobiose, salicin, arbutin, melibiose, lactose, raffinose, melezitose, inulin, glycerol, erythritol, ribitol, xylitol, l-arabinitol, d-glucitol, d-mannitol, galactitol, 2-keto-d-gluconate, d-gluconate, d-glucuronate, succinate, citrate and ethanol are assimilated. No growth occurs in d-glucosamine, d-arabinose, *myo*-inositol, d-glucono-1, 5-lactone, 5-keto-d-gluconate, dl-lactate or methanol. For the assimilation of nitrogen compounds, growth on nitrate, nitrite, l-lysine, glucosamine and d-tryptophan is positive, whereas on ethylamine, cadaverine, creatine, creatinine or imidazole, it is negative. There is no growth in the presence of 5% glucose medium with 10% NaCl and 0.01% cycloheximide. Diazonium blue B test and urease activity are positive. The maximum growth temperature is 18 °C and optimal growth temperature is 15 °C.

#### Molecular characteristics (holotype).

nucleotide sequences of ITS and nuc 28S rDNA gene sequences are deposited in NCBI GenBank under the accession numbers are MK682818 and MK682815, respectively.

#### Deposits.

***holotype***, NYNU 18562 culture was isolated from the soil of the Lianhuashan National Forest Park, Jilin Province, China, in September 2018. The holotype culture is preserved in a metabolically inactive state at Microbiology Lab, Nanyang Normal University, Henan, China. Ex-type cultures are deposited at the China Centre of Industrial Culture Collection (CICC), Beijing, China, as CICC 33355, and at the CBS Yeast Collection of the Westerdijk Fungal Biodiversity Institute, Utrecht, the Netherlands, as CBS 15868.

#### Strains studied.

NYNU 18562; paratypes: NYNU 1941 and NYNU 18410 from two different soil samples in the Maoershan National Forest Park in Yanji city, Heilongjiang Province, China.

### Mrakia arctica

Taxon classificationFungiCystofilobasidialesMrakiaceae

M. Tsuji emend. R.R. Jia & F.L. Hui

8CE9412C-181D-5E7B-A1A0-860916946E15

#### Description.

**Figure 3.**
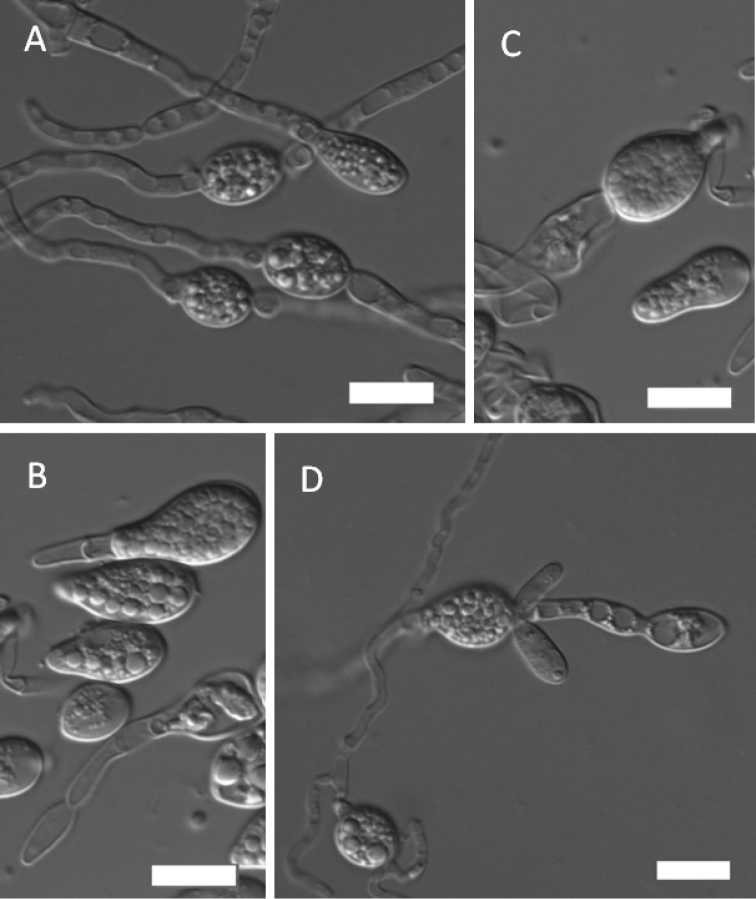
The teliospore-stage morph of *Mrakia arctica* (NYNU 18469) **A.** True hyphae with teliospores terminally and intercalary; **B/C/D.** Teliospores produced with a bud-like projection. Scale bars: 10 μm.

The yeast *Mrakia arctica* was described as an asexual species (Tsuji et al. 2018). Among soil yeasts isolated in the present study, strain NYNU 18469 collected from Liaoning provinces, formed hyphae and teliospores on CM agar (Fig. 3). In spite of the observation of sexual behavior in that species, the description of *Mrakia arctica* M. Tsuji, Mycoscience 59 (1): 57. 2018 (MB 821502) is emended below.

True hyphae with terminally and intercalarily teliospores are developed after 2 months at 10 °C on CM agar (Fig. 3A). Culture NYNU 18469 demonstrated similar morphological characteristics with those of the previously reported yeast-stage morphs, except for the yeast-forming teliospores. Teliospores are spherical, 6–12 µm in diameter, single and in short chains of 2–4 spores. Teliospores germinate by a bud-like projection (Fig. 3B, C, D). Physiologically, both asexual and sexual morphs of *M .arctica* were identical, except for a few reactions on standard growth. The asexual morph of *M .arctica* can assimilate l-sorbose and grows in the presence of 0.01% cycloheximide, and grows at 20 °C, but not for the sexual morph.

#### Deposits.

culture NYNU 18469 was isolated from the soil of the Tianhuashan National Forest Park, Liaoning Province, China, in September 2018. It is preserved in a metabolically inactive state at Microbiology Lab, Nanyang Normal University, Henan, China. Ex-type cultures are deposited at the China Centre of Industrial Culture Collection (CICC), Beijing, China, as CICC 33354, and at the CBS Yeast Collection of the Westerdijk Fungal Biodiversity Institute, Utrecht, the Netherlands, as CBS 10867.

## Discussion

*Mrakia* is currently placed in the monotypic family Mrakiaceae (Liu et al. 2015). In this study, we identified 12 isolates of *Mrakia* species from soil samples in Northeast China and characterized them using molecular sequence analyses and microscopy. As a result, a novel species was discovered among these isolates, and characterized and described as *M. panshiensis* sp. nov. Physiologically, *M. panshiensis* is different from the closely related *M. niccombsii* (Fell and Margesin 2011) in the ability to assimilate ribitol and the inability to assimilate d-glucosamine, d-arabinose and *myo*-inositol. The novel species differs from the other four members of the *M. aquatica* sub-clade in its inability to grow in 5% glucose medium with 10% NaCl and at 20 °C. Additionally, we obtained four reported *Mrakia* species, *M. aquatica*, *M. gelida*, *M. frigida* and *M. robertii*, have been reported from cold environments, such as the Alps, Alaska and Antarctica (Thomas-Hall et al. 2010; Fell 2011; Fell and Margesin 2011). These four species can be distinguished by both morphological and molecular approaches. A careful examination of isolates also resulted in the observation of the teleomorphic stage of *M. arctica*. Considering the newly described *M. panshiensis*, the genus *Mrakia* includes thirteen species. Amongst them, *M. aquatica*, *M. hoshinonis*, *M. cryoconiti* and *M. niccombsii* were described as asexual morphs. Sexual reproduction with hyphae and teliospores was observed in seven species, viz. *M. blollopis*, *M. fibulata*, *M. frigida*, *M. gelida*, *M. psychrophila*, *M. robertii*, *M. montana*, *M. stelviica* and the newly-described *M. panshiensis* sp. nov. (Fell 2011; Tsuji et al. 2018, 2019; Yurkov et al. 2020; Turchetti et al. 2020). The question of whether or not the teleomorphs of soil yeasts depend on their hosts as well as the degree of the host specificity may shed new light on the ecology of below ground microorganisms.

Most species of the genus *Mrakia* have an optimal temperature for growth of approximately 12–15 °C and are not able to grow at temperatures above 20 °C (Fell 2011; Fell and Margesin 2011). Thus these yeasts can be defined as obligate psychrophilic yeasts (Watson 1987; Raspor and Zupan 2006). Psychrophilic yeasts are noted for their ability to grow at low temperatures and even below freezing point (Panikov and Sizova 2007). Twelve *Mrakia* isolates characterized in the present study were obtained from three regions in Northeast China. The three regions are characterized by average temperatures between −2 and 10 °C. The region represents a seasonally cold environment in Northeast China. Isolation of *Mrakia* species from seasonal soils is in line with previous observations of these yeasts from cold habitats and suggests an ecological role of *Mrakia* species in extreme environments characterized by low temperatures. Frequent sources of isolation include a variety of low temperature environments, such as ice, glacier sediment, snow and meltwater (Fell 2011; Fell and Margesin 2011; Tsuji et al. 2018, 2019). However, biodiversity and distribution of psychrophilic yeasts outside cold habitats are not well known (Yurkov et al. 2020). Sylvester et al. (2015) supposed that, although *Mrakia* seems to have a competitive advantage at 10 °C, it can grow at warmer temperatures. *M. frigida* was isolated from an oak bark in different sites across the northern United States, using an enrichment protocol and incubation at 10 °C (Sylvester et al. 2015). Several species of *Mrakia* were also obtained from xylem sap of *Betula pendula* and *Carpinus betulus* in Germany, including *M. fibulata* which exhibited maximum growth temperature at 25 °C (Yurkov et al. 2020). *Mrakia aquatica*, *M. blollopis* and *M. robertii* were isolated in summer periods from urban soils in Moscow, but not from adjacent low-managed soils (Tepeeva et al. 2018). Isolation of *M. aquatica*, *M. arctica*, *M. blollopis*, *M. gelida*, *M. fibulata*, *M. frigida*, *M. robertiiM. stelviica*, *M. montana* and the newly-described *M. panshiensis* sp. nov. from these temperate climates suggests that these yeasts might be common inhabitants of diverse environments in boreal and temperate climates.

Soils were regarded as a mere reservoir for yeasts that reside in habitats above it. Our knowledge of soil yeasts is further biased towards temperate and boreal forests. The distribution of soil yeasts is determined by plant, insect and fungal hosts and vectors (Yurkov 2018). Di Menna (1966) found *Mrakia* to be the dominant yeast genus in Antarctic soils, representing 24% of the yeast species isolated in that study. In the present study, we identified 12 isolates of *Mrakia* species from forest soils collected in Northeast China, which is corresponding to 10% of the total number of isolated yeast cultures. The sampling sites were located in temperate deciduous and broadleaf forests that are commonly found in areas with warm, moist summers and mild winters. Typical soils of temperate forests are Alfisols and Spodosols along with some Histosols. Yeast communities respond to forest properties rather than to basic soil parameters even though the mechanisms underlying these effects remain unknown (Yurkov 2017). Forest management is known to influence substrate-dependent taxa, such as bryophytes and sporocarp (fruiting body) forming fungi (Ódor et al. 2006). In view of the rapid decline of many natural habitats, studies of soil yeasts in undisturbed or low managed biotopes became extremely valuable. To date, the isolation of *Mrakia* yeasts from temperate soils is limited to a few studies from Europe (Mašínová et al. 2017; Yurkov et al. 2016; Tepeeva et al. 2018). The present study demonstrated that yeasts of the genus *Mrakia* are present in temperate forest soils in Asia.

## Supplementary Material

XML Treatment for Mrakia panshiensis

XML Treatment for Mrakia arctica

## Supplementary materials

Supplementary material 1 Figures S1 and S2 Data type phylogenetic trees https://binary.pensoft.net/file/465094This dataset is made available under the Open Database License (http://opendatacommons.org/licenses/odbl/1.0/). The Open Database License (ODbL) is a license agreement intended to allow users to freely share, modify, and use this Dataset while maintaining this same freedom for others, provided that the original source and author(s) are credited.Kai-Hong Zhang, Cheng-Feng Shi, Chun-Yue Chai, Feng-Li Hui
