# Supplementary material for: Mrakia panshiensis sp. nov. a new member of the Cystofilobasidiales from soil in China, and description of the teleomorphic-stage of M. arctica
Source: MycoKeys. 2020 Oct 20;74:75–90. doi: 10.3897/mycokeys.74.53433 (PMC7591554; doi:10.3897/mycokeys.74.53433)
Supplement: Supplementary material 1 — Figures S1 and S2 [file mycokeys-74-075-s001.pdf]

Supplementary Material Files

Title:

Mrakia panshiensis sp. nov. a new member of the Cystofilobasidiales from soil in China, and description of the teleomorphic-stage of *M. arctica*

Kai-Hong Zhang, Cheng-Feng Shi, Chun-Yue Chai, Feng-Li Hui

School of Life Science and Technology, Nanyang Normal University, *Nanyang 473061*, *PR China*

Corresponding author: *Feng-Li Hui* (fenglihui@yeah.net)

Mycokeys

Author affiliations

School of Life Science and Technology, Nanyang Normal University, Nanyang 473061, PR China
*Correspondence: Feng-Li Hui
E-mail: fenglihui@yeah.net

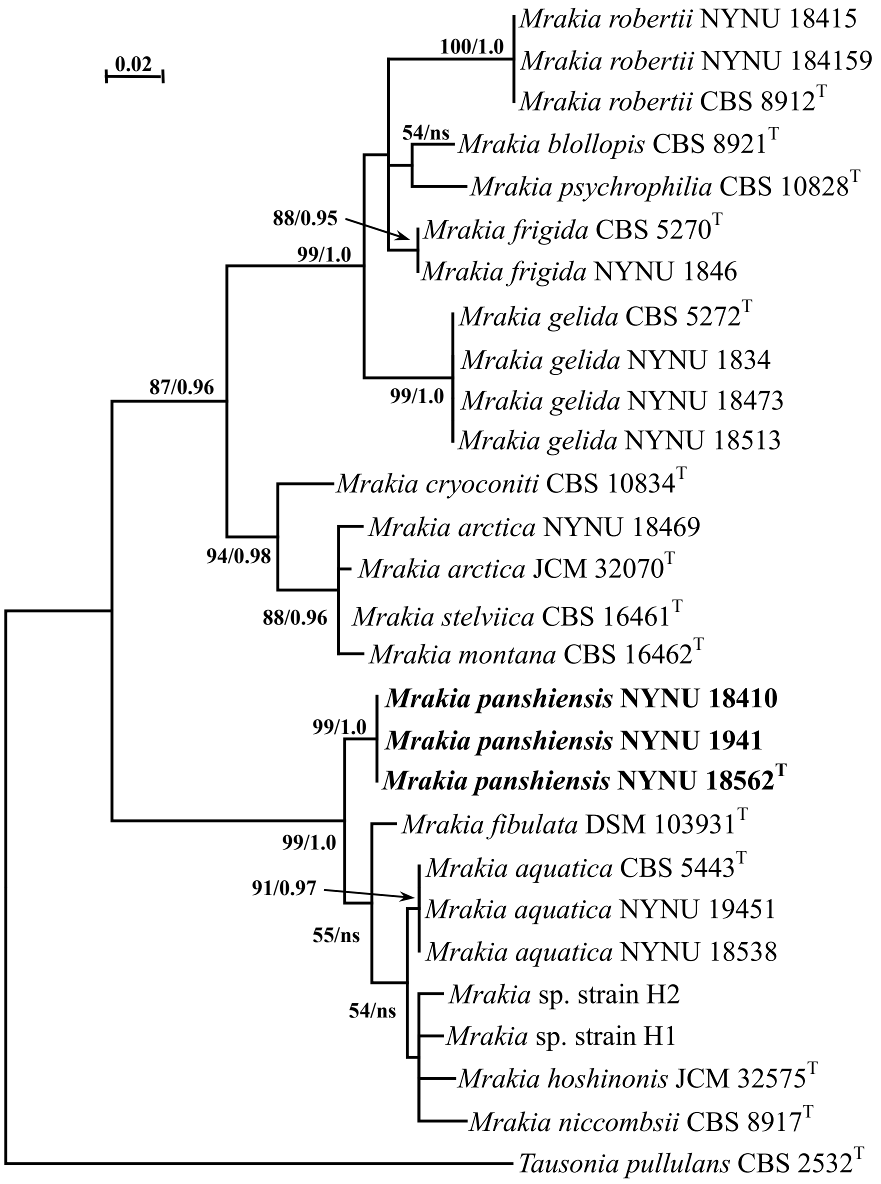

Fig. S1 Phylogenetic tree derived from the maximum-likelihood based on the ITS sequences, *Mrakia panshiensis* strains investigated in this study are highlighted in bold font. *Tausonia pullulans* CBS 2532^{T} was designated as the outgroup. The tree backbone was constructed by maximum likelihood analysis with MEGA7. Bootstrap percentages of maximum likelihood over 50% from 1000 bootstrap replicates and posterior probabilities of Bayesian inference above 0.9 are shown from left on the branches. The scale bar represents 0.02 substitutions per nucleotide.

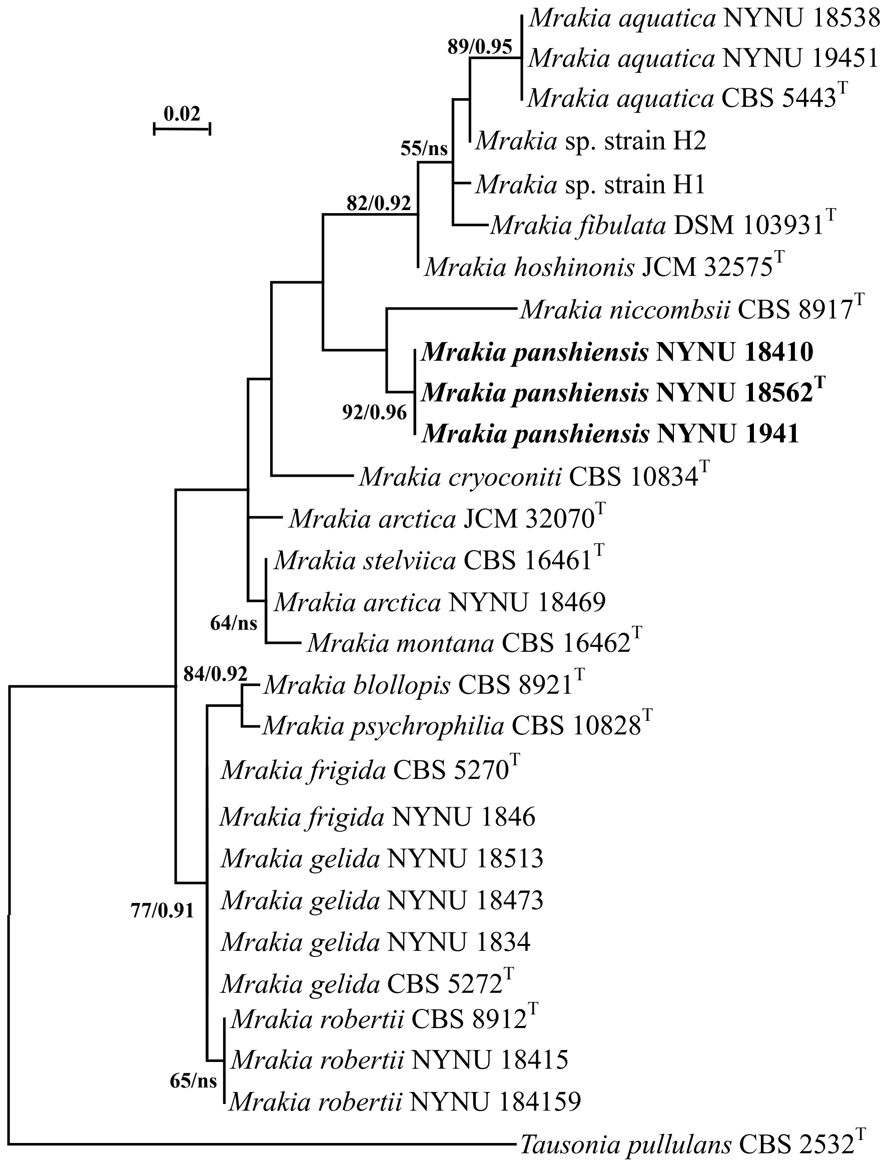

Fig. S2 Phylogenetic tree derived from the maximum-likelihood based on the the gene sequences of nuc 28S rDNA gene, *Mrakia panshiensis* strains investigated in this study are highlighted in bold font. *Tausonia pullulans* CBS 2532^{T} was designated as the outgroup. The tree backbone was constructed by maximum likelihood analysis with MEGA7. Bootstrap percentages of maximum likelihood over 50% from 1000 bootstrap replicates and posterior probabilities of Bayesian inference above 0.9 are shown from left on the branches. The scale bar represents 0.02 substitutions per nucleotide.
